# A Compact Millimeter-Wave Multilayer Patch Antenna Array Based on a Mixed CPW-Slot-Couple Feeding Network

**DOI:** 10.3390/mi15040535

**Published:** 2024-04-16

**Authors:** Kun Deng, Naibo Zhang, Guangyao Yang, Yitong Li, Ruiliang Song, Ning Liu

**Affiliations:** Beijing Research and Development Center, The 54th Research Institute of China Electronics Technology Group Corporation (CETC 54), Beijing 100070, China; dengkun@bupt.edu.cn (K.D.); y85571958@163.com (G.Y.); ytongm@163.com (Y.L.); songruiliang@hotmail.com (R.S.); liuning1512@163.com (N.L.)

**Keywords:** millimeter-wave antenna array, patch antennas, CPW feeding network, characteristic mode analysis (CMA)

## Abstract

A compact Ka-band antenna array has been proposed to realize broadband and high gain for millimeter-wave applications. The antenna array is divided into a multilayer composed of a driven slot patch layer and a parasitic patch array layer, which is excited by a mixed CPW-Slot-Couple feeding network layer. According to characteristic mode analysis, a pair of narrow coupling slots are introduced in the driven patch to move the resonant frequency of characteristic mode 3 to the resonant frequency of characteristic mode 2 for enhanced bandwidth. In this article, a 1to4 CPW-Slot-Couple feeding network for a 2 × 2 driven slot patch array is implemented, and then each driven slot patch excites a 2 × 2 parasitic patch array. Finally, a proposed 4 × 4 × 3 (row × column × layer) Ka-band antenna array is fabricated to verify the design concepts. The measured results show that the frequency bandwidth of the antenna array is 25 GHz to 32 GHz, and the relative bandwidth is 24.5%. The peak gain is 20.1 dBi. Due to its attractive properties of miniaturization, broadband, and high gain, the proposed antenna array could be applied to millimeter-wave wireless communication systems.

## 1. Introduction

Recently, the research on millimeter-wave and terahertz technologies has promoted the development of compact and miniaturized antennas [1], such as patch antennas, waveguide antennas, reflecting antennas, and metamaterial antennas. Among them, patch antennas are widely used in Ka-band satellite communication, mobile communication, and the internet of vehicles and things owing to their attractive merits, low profile, and high integration. For millimeter-wave antennas, bandwidth and gain are the most important indicators, where wide bandwidth is essential for high-speed data transmission and high gain is essential to cover spacious areas [2]. However, the traditional patch antenna still faces the following problems in its application: (1) The millimeter-wave patch antenna bandwidth is limited by its profile size; it suffers from a narrow bandwidth of less than 10%. (2) Millimeter-wave patch antenna gain is limited by the array scale; it suffers from a large loss of the feeding network. To solve the above problems, many researchers have carried out in-depth explorations into multi-mode and multilayer antenna design methods.

It is well known that by bringing two or more modes close to each other, bandwidth and gain performance would be improved to some extent [3,4,5,6,7]. A traditional method is to adopt shorting pins or vias, which would disturb the equivalent current on the radiation patch so that more resonant modes participate in the radiation. Ref. [8] proposed a wideband patch antenna capable of realizing multiple directional patterns; the antenna adopts a slot coupling feed, and the TM_20_/TM_21_ modes are combined by loading shorting pins on the four corners; the bandwidth is 7.3% and the peak gain is 9.8 dBi. In Ref. [9], a circle of shorting pins is loaded on the antenna to excite the TM_01_ mode resonant frequency, and the rectangular slot is used to reduce the TM_12_ mode resonant frequency, so that the two modes are close to each other, and the final antenna frequency bandwidth is extended to 9% and the peak gain is 6.5 dBi. Although the above technique can make multiple modes radiate together, due to the difficulty of impedance matching, the antenna radiation efficiency is low, which limits its practical application.

As for multilayer technology, its essence involves increasing the antenna profile height and forming multiple resonators to improve the radiation performance [10,11,12,13]. Ref. [14] proposed an 8 × 8 dual-polarized millimeter-wave antenna array, which adopts a two-layer SIW feeding network to excite multiple resonant modes to expand the frequency bandwidth; the measured results have a bandwidth of 14.6% (36.8–42.6 GHz) and a peak gain of 25.8 dBi. Ref. [15] proposed a 4 × 4 CP patch antenna array, which adopts a double-layer substrate; due to the increase in profile height, the bandwidth is extended. The measured results show that the bandwidth is 16% (41.9–49.1 GHz) and the array gain is greater than 17 dBi. In addition, multilayer antennas can realize some specific radiation requirements, such as circular polarized, multi-polarized, multi-beam, etc. However, conventional multilayer antennas rely on a complex feeding network, resulting in low efficiency [16].

**Figure 1:**
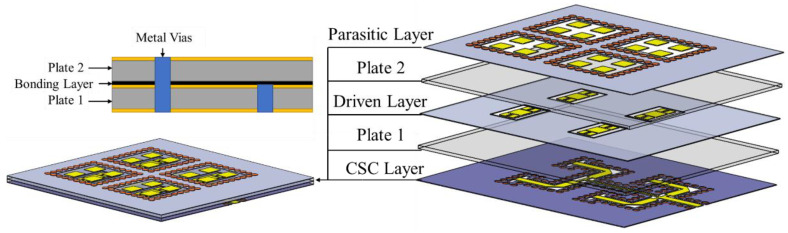
Antenna array 3D diagram.

In this paper, a novel multilayer patch antenna array based on a mixed CPW-Slot-Couple (CSC) feeding network to realize a wide bandwidth and high gain for Ka-band applications is presented, as shown in Figure 1. The proposed antenna array is divided into three layers, which are the feeding network layer, the driven slot patch antenna layer, and the parasitic patch array layer from the bottom up. According to characteristic mode analysis, a square patch with a pair of coupling slots is stacked together as a driven patch; then, a 1to4 CSC feeding network for a 2 × 2 driven slot patch array is implemented, and each driven slot patch excites a 2 × 2 parasitic patch array. Finally, a proposed 4 × 4 × 3 (row × column × layer) antenna array is fabricated and measured to verify the design concepts. The measured results show that the frequency bandwidth of the antenna array is 25 GHz to 32 GHz, and the relative bandwidth is 24.5%. The peak gain was 20.1 dBi.

## 2. Design of the Proposed Antenna Array

In this section, the design flow from all layers of the proposed antenna array will be illuminated in detail. Firstly, according to the principle of multi-mode excitation, a square patch and a pair of coupling slots are stacked together as a driven patch. Then, multilayer radiation analysis is applied to guide the design of the parasitic patch array. Finally, we present the 1to4 CSC feeding network to realize a wide bandwidth and high-gain antenna array.

### 2.1. Driven Slot Patch Antenna Layer

**Figure 2:**
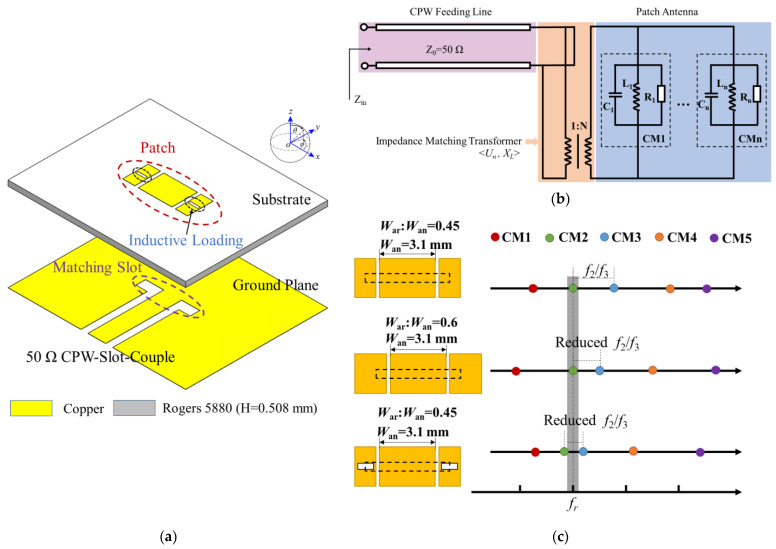
(**a**) Schematic topology of the driven slot patch antenna element; (**b**) equivalent circuit model of the proposed antenna element; (**c**) graphical demonstration the antenna size and CM frequency.

The driven antenna element characteristic mode analysis and design processes are presented in Ref. [17]. The antenna is composed of a nonuniform patch with a pair of coupling slots on a low-dielectric-constant high-frequency substrate (Rogers 5880, USA, ROGERS Corporation, h = 0.508 mm, *ε_r_* = 2.2, tan*δ* = 0.0009), as shown in Figure 2a. The equivalent circuit is shown in Figure 2b, which could be characterized by a series of characteristic modes (CMn). An inductively coupled excitation (ICE), such as conductor circles or slots, could move a higher-order CM to a lower-order desired CM to enhance the bandwidth of the antenna. As shown in Figure 2c, two matching slots adopted on the patch are used for impedance matching, which can help enhance the bandwidth and gain by shifting CM3 closer to CM2.

**Figure 3:**
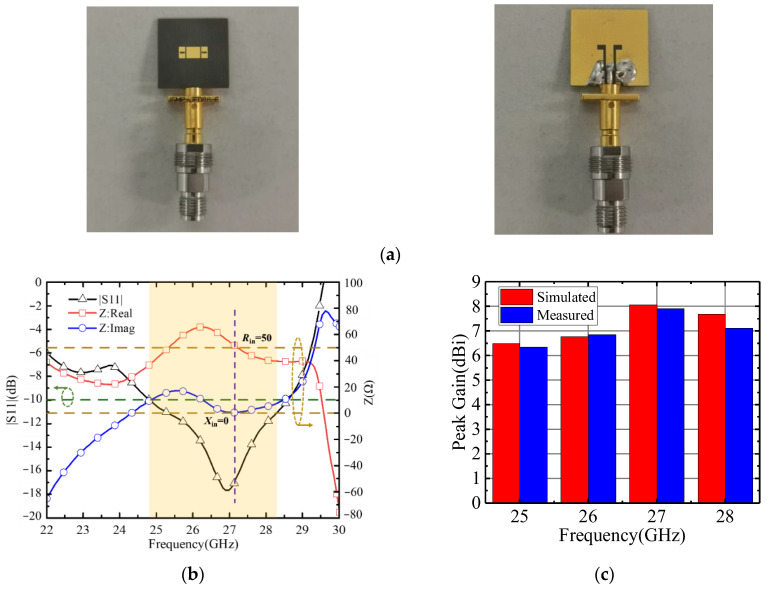
(**a**) Photograph of the fabricated sample; (**b**) the reflection coefficients and matched impedance; (**c**) simulated and measured gain of the proposed antenna.

The optimized parameters of the proposed antenna element were simulated in detail, and the final fabricated and measured results are shown in Figure 3. Figure 3a shows the top- and bottom-view photographs of a fabricated prototype, with a compact size of 7.23 × 2.6 × 0.508 mm^3^. The impedance (*Z*_in_ = *R*_in_ + *jX*_in_) and reflection coefficients (S11) are shown in Figure 3b. The antenna impedance is well matched in the range of 25 GHz to 28 GHz, where S11 < −10 dB, and it obtains *R*_in_ = 50 Ω and *X*_in_ = 0 at 27 GHz. Figure 3c shows the simulated and measured realized gain versus frequency. The measured peak gain is 7.9 dBi; the enhanced gain characteristics are thanks to the successful implementation of the nonuniform patch [17].

### 2.2. Parasitic Patch Antenna Array Layer

**Figure 4:**
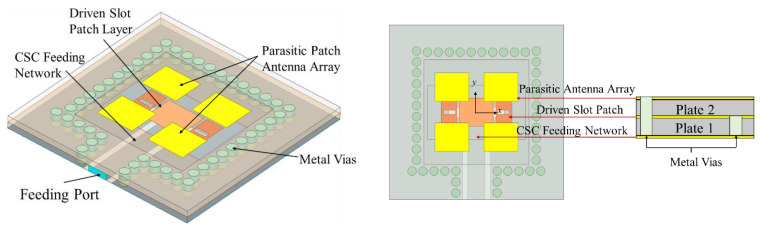
Schematic diagram of the multilayer antenna subarray.

**Figure 5:**
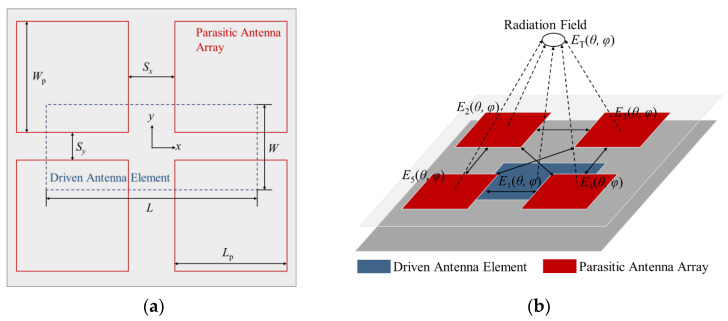
Schematic diagram of radiation field analysis of the multilayer antenna subarray: (**a**) structure diagram; (**b**) radiation field diagram.

To improve the gain, the parasitic patch antenna array layer was designed based on the driven antenna element above; Figure 4 shows a schematic diagram of the multilayer antenna subarray, which ignores the bonding layer. The whole antenna subarray consists of three layers of metal plates and two layers of Rogers 5880 plates. From bottom to top, the first metal plate layer is the CSC feeding network, which also serves as the ground for the second layer of the driven antenna element; the third metal plate layer is the parasitic antenna array, and four patches form a subarray, which are couple-fed by a driven antenna element; each layer of the antenna subarray is connected by metal vias, which could reduce the mutual coupling between the driven antenna element and the parasitic antenna array, as well as suppressing surface waves [18]. Figure 5a shows the main view of the antenna subarray, and Table 1 gives each dimension parameter.

As shown in Figure 5b, the radiation field of the antenna subarray (*E_T_*) can be regarded as a superposition field under the mutual coupling between the driven antenna element, *E_d_* = *E*_1_, and the parasitic antenna array, *E_P_* = (*E*_2_||*E*_3_||*E*_4_||*E*_5_), which could be described by the mutual coupling coefficient, *K_C_*:

(1) $$E_{T}(\theta ,\varphi )=E_{d}(\theta ,\varphi )+K_{C}E_{P}(\theta ,\varphi )$$

**Figure 6:**
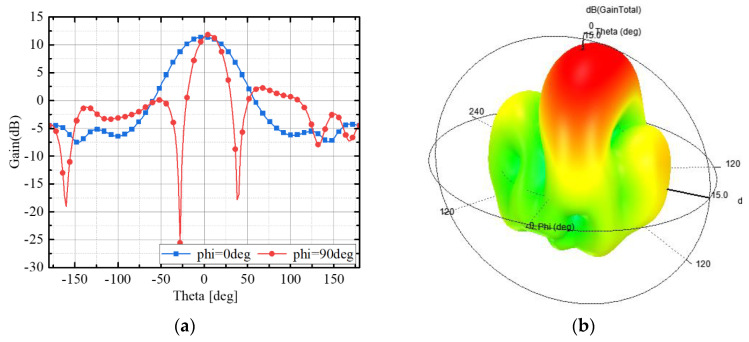
Simulation results of antenna subarray radiation pattern at 30 GHz: (**a**) gain; (**b**) directional diagram.

We simulated the proposed antenna using the HFSS 2022 software. Figure 6 shows the radiation pattern of the proposed antenna subarray on the phi = 0° and phi = 90° planes at 30 GHz. It can be seen from the figure that the sidelobe is suppressed below −10 dB. Since neither the driven antenna element nor the parasitic array are centrosymmetric, the pattern of the 0° and 90° planes is not similar. The radiation pattern has better directivity in the 90° plane and a larger beamwidth in the 0° plane. The simulated peak gain of the proposed antenna subarray is 12.1 dBi at 30 GHz.

### 2.3. CSC Feeding Network Layer

The design processes of the driven layer and parasitic layer are described in detail above. In this paper, the proposed multilayer antenna array is obtained with a compact size of 4 × 4 × 3 (row × column × layer); therefore, it is necessary to design a feeding network for the 2 × 2 proposed multilayer antenna subarray (as shown in Figure 4). If a larger array of antennas is required, it can be expanded on this basis.

**Figure 7:**
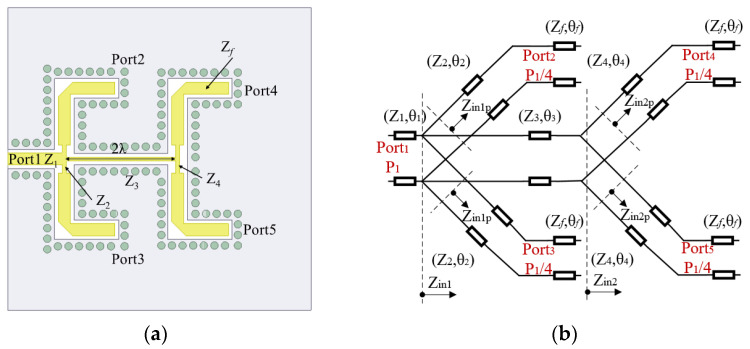
CSC feeding network: (**a**) schematic diagram; (**b**) equivalent circuit diagram.

A CPW-Slot-Couple (CSC) feeding network is implemented in the bottom layer, which is a 1to4 series–parallel feeding network. Figure 7 shows the schematic diagram and equivalent circuit diagram. Port 1 is the input port, and ports 2–5 are the output ports, which are, respectively, connected to the driven slot antenna elements. *Z*_1_ and *Z_f_* are the characteristic impedance of the input and output ports, respectively, which are both 50 ohms. Among other things, it also contains two quarter-wavelength impedance matching lines, *Z*_2_ and *Z*_4_, and a two-wavelength transmission line, *Z*_3_; all lines are enclosed by metal vias. According to the equivalent circuit impedance matching principle, *P*_1_ represents the input power, *θ* represents the electrical length of each section of the transmission line, and *Z*_*in*1_, *Z*_*in*2_, *Z*_*in*1*p*_, and *Z*_*in*2*p*_ represent the equivalent input impedance; the impedance equation of each section of the transmission line is expressed as follows:

(2) $$\begin{array}{cc} Z_{3}=Z_{in2}=Z_{in2p}∥Z_{in2p}; & Z_{4}^{2}=Z_{in2p}Z_{f} \end{array}$$

(3) $$\begin{array}{cc} Z_{1}=Z_{in}=Z_{3}∥Z_{in1p}∥Z_{in1p}; & Z_{2}^{2}=Z_{in1p}Z_{f} \end{array}$$

(4) $$Z_{2}=Z_{4}=2Z_{f}$$

The characteristic impedance of each transmission line can be obtained by the above equation, and the corresponding line width and length can be calculated. The impedance and size values of each transmission line are shown in Table 2.

**Figure 8:**
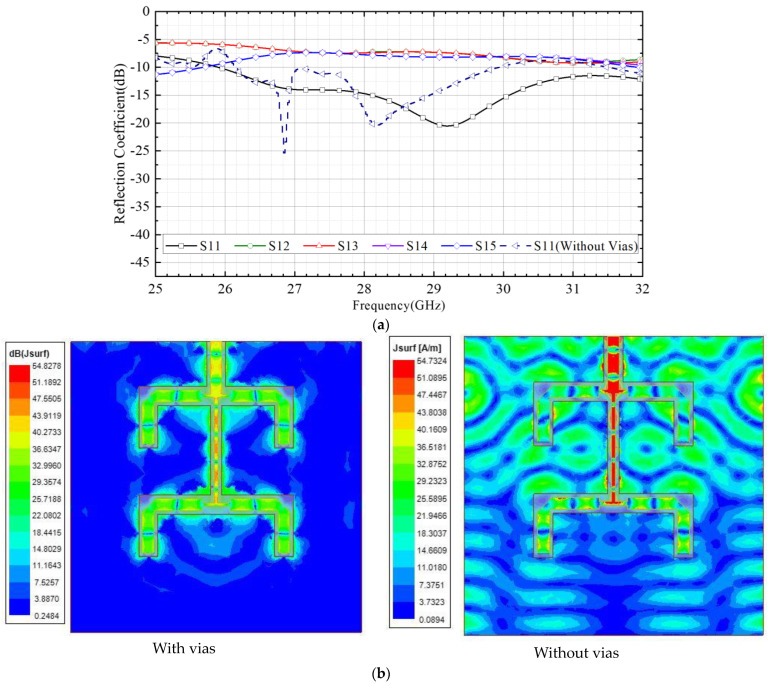
(**a**) S parameter of the CSC feeding network; (**b**) surface current distribution diagram at 30 GHz of the feeding network with or without vias.

According to the calculated size of the feeding network, a 3D model is established in the electromagnetic simulation software HFSS. The simulated S-parameter curve of the feeding network is shown in Figure 8. It can be seen from the figure that in the frequency range of 26 GHz to 31 GHz, the reflection coefficient of port 1 |S11| > 10 dB, and the reflection coefficients of port 1 to 2, 3, 4, and 5 (|S12-5|) are in the range of 6–8 dB, which meets the design requirements of the 1to4 feeding network. The simulated S11 curve of the feeding network without vias is also shown in Figure 8a. It can be seen that the S11 of the feeding network without vias deteriorates rapidly at frequencies >30 GHz. As shown in Figure 8b, it can be seen from the surface current distribution diagram that at 30 GHz, the metal vias can effectively reduce the surface current distribution, reduce energy loss, and improve the isolation between each feed port.

## 3. Physical Dimensions and Fabrication

**Figure 9:**
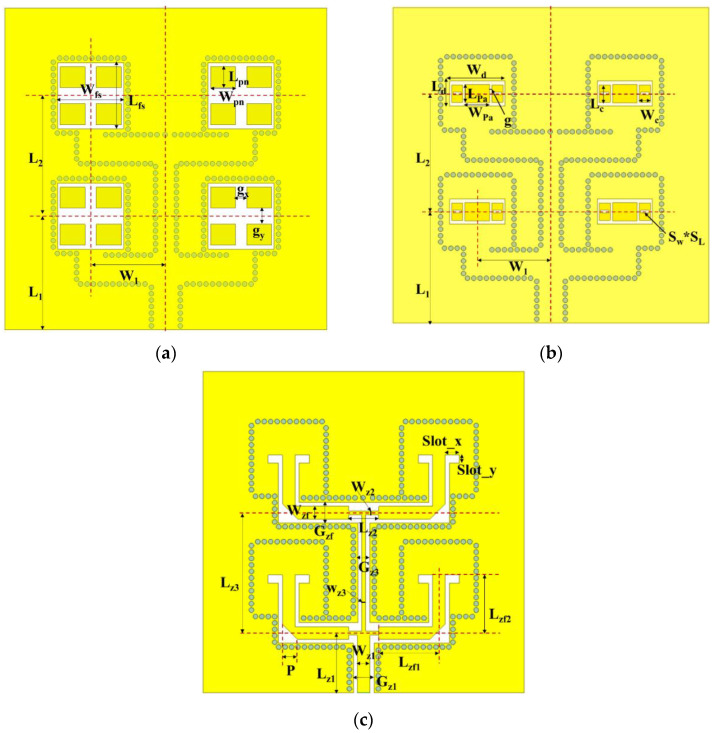
Schematic diagram of antenna layers: (**a**) parasitic antenna array layer; (**b**) driven slot patch antenna layer; (**c**) CSC feeding network layer.

Through the above analysis, the physical dimensions of the proposed multilayer antenna array are shown in Figure 9 and Table 3.

**Figure 10:**
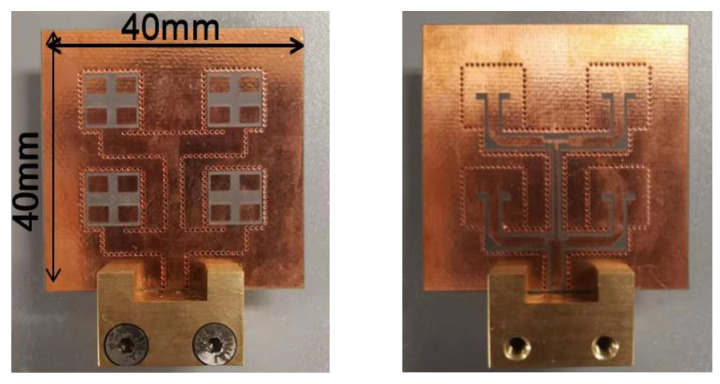
The fabricated antenna array.

In this paper, the multilayer antenna array consists of three layers of metal plates and two layers of Rogers 5880 plates, and multiple plates are connected by bonding layers (FR28-0040-50, *ε*_r_ = 2.74, *h* = 0.1 mm). From bottom to top, the first metal plate layer is the 1to4 CSC feeding network, which also serves as the ground for the second layer of the 2 × 2 driven slot patch antenna array; the third metal plate layer is the 4 × 4 parasitic antenna array, which is couple-fed by a driven antenna element; each layer of the antenna array is connected by metal vias. The fabricated antenna array is shown in Figure 10.

## 4. Measured Results and Discussion

In this section, the proposed 4 × 4 × 3 (row × column × layer) multilayer antenna array is measured and discussed. The reflection coefficient was measured by a vector Network Analyzer (R&S ZVA50, Rohde & Schwarz, Munich, Germany), and the radiation patterns were measured by a compact antenna test range.

### 4.1. Measured Results

**Figure 11:**
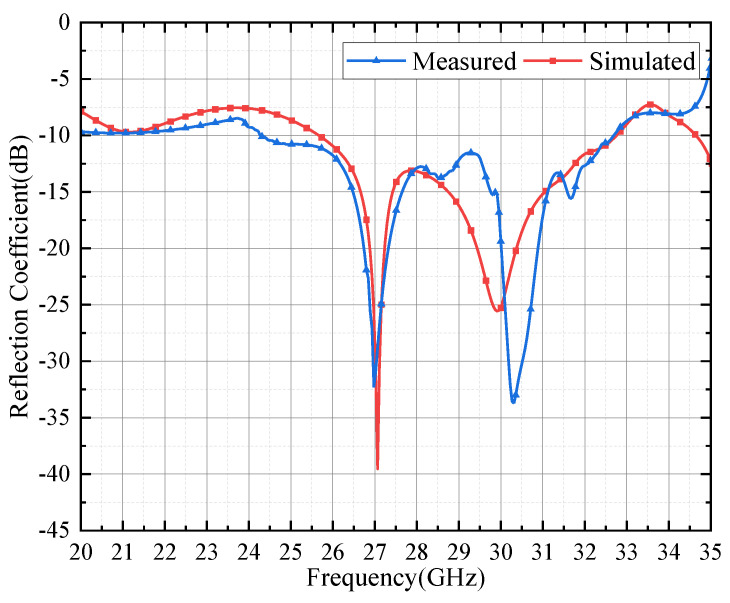
Simulated and measured results of reflection coefficient.

The simulated and measured reflection coefficients are shown in Figure 11. We simulated the proposed antenna through the HFSS software. There are two peaks in the reflection coefficient curve with the central frequencies of 27 GHz and 30.2 GHz, which are derived from the driven layer and parasitic layer, respectively. It can be observed that the simulated and measured results agree well with each other at low frequencies, but the measured results are greater than the simulated one in the range of 29 GHz to 30 GHz, and the resonant point near 30 GHz moves to the high-frequency band, which may be due to the influence of mutual coupling between parasitic patches in the measured field. In the frequency range of 25 GHz to 32 GHz, the reflection coefficient of the proposed antenna array |S11| < −10 dB, and the relative bandwidth is 24.5%.

### 4.2. Radiation Patterns and Gains

**Figure 12:**
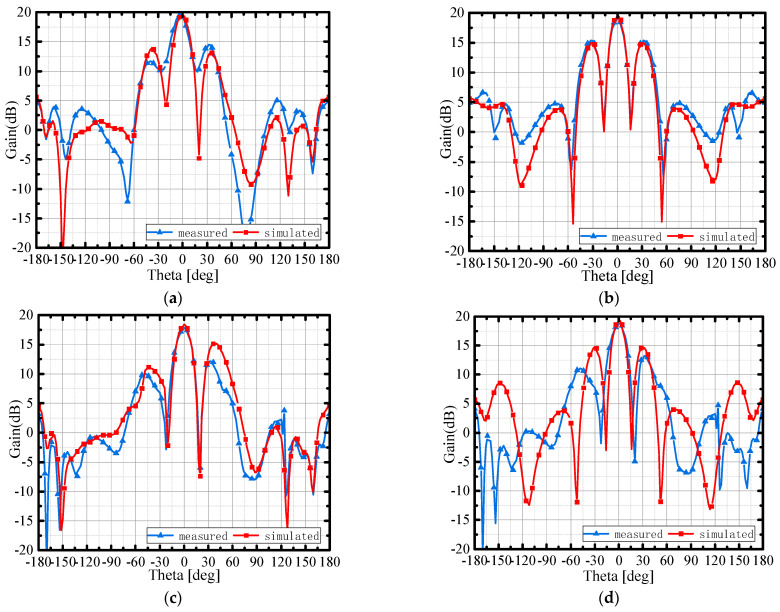
Radiation patterns: (**a**) phi = 0° plane at 27 GHz; (**b**) phi = 90° plane at 27 GHz; (**c**) phi = 0° plane at 30 GHz; (**d**) phi = 90° plane at 30 GHz.

Figure 12 shows the radiation patterns in the phi = 0° and 90° planes at two resonant points of 27 GHz and 30 GHz.

At the frequency of 27 GHz, the maximum simulation gain in the main radiation direction is 19 dB, and the measured peak gain is 19.6 dB. At the frequency of 30 GHz, the simulation result is 16 dB, and the measured peak gain is 18.8 dB. It can be observed that the results of the simulated and measured peak gains agree well in the main beam direction. However, there are strong minor lobes because the distance between the parasitic subarrays (as shown in Figure 4) is up to 1.45λ, which is greater than one λ, where λ is the wavelength of 30 GHz. The strong minor lobes can be suppressed by a more compact feeding network to reduce the antenna unit space. Generally speaking, there is a stable radiation pattern of the antenna array.

Table 4 shows the gain and radiation efficiency of each frequency point of 25–32 GHz. It can be seen that the overall gain of the antenna is greater than 18 dB, the radiation efficiency is greater than 30%, and the maximum radiation efficiency within the frequency bandwidth is 58%. Compared to the efficiency of the same type of antenna in the literature, the efficiency of the array antenna designed in this paper is slightly insufficient. This is because, although the design of the laminated element subarray can effectively expand the frequency bandwidth of the antenna, it also aggravates the energy loss of the multilayer plate feed.

### 4.3. Discussion

Table 5 is a performance comparison between the proposed multilayer antenna array and other reported milliliter-wave antenna arrays. In terms of size and BW, the profile size of the antenna designed in this paper is only 0.1016λ, which is one-quarter to one-half of the same type of antenna, and the frequency bandwidth and gain are equal to or higher than others. However, compared with the efficiency, the proposed antenna is slightly insufficient, which can be explained by the fact that, although the multilayer can effectively expand the BW, it also aggravates the energy loss of the multilayer plates. Therefore, compared with other millimeter-wave antenna, the proposed one has attractive properties of miniaturization, broadband, high gain, and easy fabrication and integration.

## 5. Conclusions

In this paper, a novel compact Ka-band multilayer patch antenna array based on a mixed CPW-Slot-Couple feeding network is proposed. The array is divided into three metal layers, and from the bottom up, the first metal layer is a CSC feeding network for the second metal layer of the driven slot patch array. At the top is the parasitic layer, which is couple-fed by a driven layer to realize a stable radiation pattern. Then, a 4 × 4 × 3 (row × column × layer) multilayer antenna array is fabricated to verify the design concepts, exhibiting good agreement between the simulated and measured results. Finally, the measured results show that the frequency bandwidth of the antenna array is 25 GHz to 32 GHz, and the relative bandwidth is 24.5%. The peak gain is 20.1 dBi. Compared with other reported millimeter-wave antenna arrays, the proposed antenna has attractive properties of miniaturization, broadband, high gain, and easy fabrication and integration. This paper has several limitations, which should be improved in future research: First, the analysis of the effect of antenna directivity on gain is insufficient, which is inaccurate for the reflection coefficient and radiation efficiency. Radiation field analysis at specific frequencies should be considered. Secondly, multilayer dielectric loss and coupling feed loss are the main causes of low radiation efficiency; they should be taken into account in the theoretical model.

## Figures and Tables

**Table 1:** Physical dimensions of the multilayer antenna array.

**Symbol**	*W*	*L*	*W_p_*	*L_p_*	*S_x_*	*S_y_*
**Value**	7.23 mm	2.6 mm	3.1 mm	2.6 mm	1.4 mm	2 mm

**Table 2:** Physical dimensions of the CSC feeding network.

	(*Z*_1_, *θ*_1_)	(*Z*_2_, *θ*_2_)	(*Z*_3_, *θ*_3_)	(*Z*_4_, *θ*_4_)	(*Z_f_*, *θ_f_*)
Impedance	50 Ω	50 Ω	100 Ω	100 Ω	50 Ω
Elec. Length	360°	90°	360°	90°	654.5°
Width	1.578 mm	0.484 mm	0.484 mm	0.484 mm	1.578 mm
Length	7.2 mm	1.87 mm	14.96 mm	1.87 mm	13.09 mm

**Table 3:** Physical dimensions of the antenna (unit: mm).

**Parasitic antenna array layer**								
**Symbol**	W_fs_	L_fs_	W_pn_	g_y_	g_x_	L_pn_		
**Value**	8.2	8.2	3.1	2	1.4	2.6		
**Driven slot patch antenna layer**								
**Symbol**	W_1_	L_1_	L_2_	W_d_	L_d_	W_Pa_	L_Pa_	g
**Value**	7.48	14.68	14.96	7	3.2	3.12	2.32	0.29
**Symbol**	W_c_	L_c_	S_W_	S_L_				
**Value**	1.4	2.16	1.03	0.36				
**CSC feeding network layer**								
**Symbol**	W_z1_	G_z1_	L_z1_	W_z2_	L_z2_	W_z3_	G_z3_	L_z3_
**Value**	1.578	2.578	7.2	0.484	3.74	0.484	1.484	14.96
**Symbol**	W_zf_	G_zf_	L_zf1_	L_zf2_	Slot_x	Slot_y	P	
**Value**	1.578	2.578	5.61	7.48	1.77	1.01	1.98	

**Table 4:** The measured gains of the antenna array.

Frequency	25 GHz	26 GHz	27 GHz	28 GHz	29 GHz	30 GHz	31 GHz	32 GHz
Gain (dB)	16.7	18.3	19.6	20.1	19.4	18.8	17.9	17.5
Efficiency	31%	42%	56%	58%	51%	47%	38%	35%

**Table 5:** Performance comparison between the proposed and other reported millimeter-wave antennas.

Ref.	Technical Method	Size (λ)	Array Scale	Freq. (GHz)	BW (%)	Peak Gain (dB)	Efficiency RAD. (%)
[19]	Parasitic patches + SIW	6.8 × 4.5 × 0.21	4 × 4	28	18.6	18.2	65
[20]	Rotary feed + SIW	4.8 × 4.8 × 0.49	4 × 4	30	14.1	19.5	77
[21]	Spiral antenna + SIW	2.4 × 2.0 × 0.4	4 × 4	30	22	15.2	54.9
[22]	Electromagnetic dipole	6.8 × 6.1 × 0.49	8 × 8	30	18.2	26.1	75
[23]	Slot antenna + CPW	12 × 12.7 × 0.14	16 × 16	20	15.9	25.9	--
[24]	Resonant Cavity	5.57 × 8.57 × 2.36	2 × 2	25	15.15	7.69	62
[25]	Short probe + SIW	3.6 × 3.6 × 0.508	1	28	3.4	2.3	82
[26]	Slot + SIW	4.48 × 4.48 × 2.575	4 × 4	30	44.62	7.25	--
[27]	SIGW + Air cavity-backed	3.8 × 3.8 × 2.361	2 × 2	26	19	5.51	65
[12]	ESIW + Slot	2.6 × 4 × 1.016	2 × 2	28.25	2.4	12	85
This paper	Multilayer antenna + CSC feeding network	4 × 4 × 0.1016	4 × 4	30	24.5	20.1	58

## Data Availability

The original contributions presented in the study are included in the article, further inquiries can be directed to the corresponding author.
